# SOCIAL PARTICIPATION AND GENDER DISPARITY ON DEPRESSIVE SYMPTOMS AMONG INDONESIAN ELDERLY

**DOI:** 10.1093/geroni/igz038.585

**Published:** 2019-11-08

**Authors:** Riska D Astuti, Bondan Sikoki

**Affiliations:** 1 SurveyMETER institute, Yogyakarta, Indonesia, Indonesia; 2 SurveyMETER, Yogyakarta, Indonesia, Indonesia

## Abstract

A voluminous literature empirically proves the significant relationship between social participation and elderly mental health. Further analysis discovers the different behavior between rural and urban elderly. Despite the importance of taking into account regional inequality, gender disparity which explains the inconsistent empirical results across countries, is sometimes negligible from the discussion. This study aims to investigate the link of social participation in voluntary activity and community regular meeting to depressive symptom among the elderly in Indonesia. Separated analysis based on gender is also conducted to examine the extent to which social activities could explain the depressive symptom disparity between male and female elderly. Data from Indonesia Family Life Survey 2007 and 2014 were analyzed using logistic regression. Sample of 2994 and 2917 respondents aged 60 and over in 2007 and 2014 respectively are combined as pooled cross-sectional data instead of panel data to deal with the large reduction of sample size due to mortality. To minimize the potential endogeneity, covariates are included in the model such as residence location, living arrangement, socio-economic status, and health condition. The results indicate that economic condition, chronic disease, and difficulty on instrumental activity daily living (IADL) play a significant role in depressive symptom among Indonesian elderly, regardless of the gender. Surprisingly, social participation that is widely believed in strengthening mental health is statistically significant for female sample only. Moreover, the contrast sign of voluntary participation and community regular meeting coefficients indicate a special behavior between these two activities.

